# The Evolution of Physical Laws and the Entropic Measure of Time

**DOI:** 10.3390/e28070792

**Published:** 2026-07-13

**Authors:** Leonid M. Martyushev

**Affiliations:** 1 Technical Physics Department, Ural Federal University, 19 Mira St., 620002 Ekaterinburg, Russia; leonidmartyushev@gmail.com; 2 Institute of Industrial Ecology, Russian Academy of Sciences, 20 S. Kovalevskaya St., 620219 Ekaterinburg, Russia

**Keywords:** time, epistemology, laws of nature, evolution, maximum entropy production, constructivism, entropic measure of time

## Abstract

The traditional paradigm of natural science treats the laws of nature as eternal and immutable. This review examines a powerful alternative tradition that views these laws as historically evolving and constructed entities, tracing this shift from ancient roots to evolutionary epistemology, radical constructivism and physics. Specifically, it provides a chronological analysis of how ideas about the variability of laws developed from ancient Greek philosophy through Enlightenment thinkers to contemporary physicists like Ilya Prigogine and Lee Smolin. We address the resulting methodological crisis—where different branches of science optimize their own laws and isolate from one another—by proposing a strict hierarchical framework. Under this method, invariant basic concepts are strictly separated from flexible models. Crucially, the Entropic Measure of Time (EMT) is presented as the central operational tool. By defining time through entropy production, EMT enables the deductive derivation of physical laws from specific models, restoring a unified, cohesive structure to modern science and offering a robust strategy to counteract the fragmentation of scientific disciplines.

## 1. Introduction

The idea that the world is governed by eternal, immutable laws, which a scientist discovers (reveals) with greater or lesser difficulty, is a classical representation of modern natural science. This point of view originated as far back as antiquity [1,2,3]. Thus, Pythagoras believed that everything is based on number, the harmony of the cosmos is mathematical, and it is eternal. We do not invent mathematics; we discover it within the structure of being. Heraclitus of Ephesus introduced the concept of the *Logos*—a universal law, an objective order to which even the “changeable” fire is subject. Democritus, the founder of atomism, argued that everything in the world happens by necessity: no thing arises without a cause, but everything arises on some ground and by virtue of necessity. For Plato, the laws of nature are not merely regularities of the material world, but reflections of higher, eternal entities—ideas (*eide*), which are objective, eternal, and exist in a “higher reality” even before the emergence of the Universe. Aristotle believed that although things within the world change, the Cosmos itself and the fundamental principles of nature are eternal [1,2,3].

In the Middle Ages, the belief in an immutable order established by God or Reason predominated. In the Early Modern period, Descartes viewed the world as a gigantic, well-functioning mechanism (a clock) wound up by God [4]. The laws of motion of matter are immutable and knowable through reason. Newton solidified the image of the Universe as a system obeying strict mathematical laws (the law of universal gravitation, etc.) [5]. Finally, Pierre-Simon Laplace formulated the extreme point of view—“Laplace’s Demon”. If a certain intellect knew the position and velocity of all particles in the Universe at a given moment, it could unerringly predict the entire future and reconstruct the entire past [6].

Despite the revolution in physics at the beginning of the 20th century, many of its founders remained “classics” in spirit. They believed that a strict mathematical harmony lay hidden behind the apparent chaos. The father of quantum theory, Planck, was a deeply believing person—both in the religious and the scientific sense. According to him, an external world exists independently of us, and it obeys absolute laws. For Planck, a scientist is one who humbly attempts to decipher the “code” written by the Creator (or Nature) [7]. Einstein believed in the “rationality” of the Universe; the laws of physics must be immutable, deterministic, and independent of the observer. His famous phrase, “God does not play dice,” expressed a protest against the idea that pure chance lies at the foundation of the world [8].

The established traditional view of the laws of nature, briefly presented here, possesses undeniable merits. This is, above all, the predictability and objectivity of the world: truth exists, and it is uniform for all researchers regardless of time and location in the Universe. Belief in the existence of a single “answer” provides a powerful stimulus for knowledge, compelling scientists to search for (discover) what is yet unknown (for instance, a formula unifying all the forces of nature—the so-called theory of everything).

Despite the described mainstream, an opposite point of view regarding the laws of nature exists. It is less known, especially among natural scientists, despite the fact that the origins of this direction also emerged in ancient philosophy, and the development of these ideas has been occurring with increasing force in recent times. This point of view is highly interesting and non-trivial; it is occasionally expressed by very well-known scientists. A brief review of works dedicated to this alternative and extremely multifaceted perspective is the purpose of this article. The connection between entropy and time, which has been recently discussed, including on the pages of this journal [9,10,11,12], will also be addressed in this review.

## 2. The Origins of the Idea of Variability of the Laws of Nature in Ancient Greek Philosophy

In the history of Western thought, ancient Greece is traditionally perceived as the cradle of the idea of an eternal and immutable order built upon invariable principles. However, parallel to the line of Plato and Aristotle, there existed an intellectual tradition that viewed the world as a process where not only things change, but also the very principles of their existence [1,2,3].

First of all, it is necessary to mention Anaximander (c. 610–540 BC), the ancient Greek philosopher from Miletus [2,3]. He is precisely the one to whom we owe the idea of the existence of laws in nature and the necessity of searching for them. His reasoning was as follows: harmony in society arises when laws exist and are followed. In nature, we observe harmony; therefore, the world around us follows certain harmonizing laws. Since these laws exist, it is important to find them. He established the goal of discovering laws, which natural science follows to this day. In his thought, one cannot yet trace the idea that since social laws are adjusted as society develops, something similar might occur with the laws of nature during its evolution.

The Sophists shifted the emphasis from the search for “absolute truth” to human perception and social practice [2,3]. For instance, Protagoras (c. 490–c. 420 BC) was the first to transfer the center of gravity of the universe from the cosmos to the human being. “Man is the measure of all things”: this thesis implies that the laws of nature do not exist in isolation from the observer. If for one person the wind is cold, and for another it is warm, nature changes its properties depending on the state of the one who perceives it. Protagoras believed that there are two opposite opinions about every single thing. Consequently, a “law” might be one thing today, and completely another tomorrow (with a change in circumstances or the viewpoint of society). For him, laws are not eternal truths, but agreements that are useful at a given moment. If an agreement ceases to work, the “law” changes.

Empedocles (c. 494–c. 434 BC) proposed one of the earliest models where the fundamental forces of the Universe do not act simultaneously at full strength [2,3]. The history of the world is a cycle. In the era of Love (*Philia*), certain principles operate (synthesis, unity), while in the era of Strife (*Neikos*), others dominate (decay, differentiation). In different eras, a different force prevails. This means that the “rules of the game” in the cosmos change: the world either merges into a single sphere, where no separate things exist, or disintegrates into chaos. Order (law) here is not static but depends on the phase of the cycle. Empedocles described transitional periods when the laws of life formation were different, and “monsters” (heads without shoulders) appeared in the world, which were later displaced by more stable forms.

Epicurus (341–270 BC) and his follower Lucretius Carus (c. 99–c. 55 BC) built their physics on two pillars: infinite empty space and an infinite number of atoms [2,3,13]. The logic of the Epicureans was as follows: if the Universe is infinite, then all possible combinations of atoms are realized within it. The Epicureans believed that a multiplicity of worlds exists in the infinite Universe. In each of them, the laws of physics may differ depending on exactly how the atoms collided and interlocked in that particular region of space. An important feature of Epicurean physics was the *clinamen*. This is a small, random deviation of an atom from a straight path during its motion, occurring without any cause. This implies that indeterminism (the absence of strict predetermination) lies at the foundation of nature. The introduction of the spontaneous swerving of atoms destroyed the idea of fatalism. If an atom can alter its trajectory without a cause, it means that the laws of nature do not possess an absolute character.

Thus, already in ancient Greece, an intellectual framework emerged for understanding the world as an evolving system. Their ideas regarding randomness, relativism, and the cyclical alternation of phases anticipated modern scientific discussions about the possibility of changing physical constants, laws, and the Multiverse theory.

## 3. Ideas of Variability: Between Antiquity and the Nineteenth Century

For more than a millennium and a half, European thought practically did not return to the idea of changing the laws of nature. This intellectual lull was conditioned by two fundamental reasons [14,15]:

1. *Theological determinism.* In the monotheistic picture of the world (Christianity, Islam), God was understood as the perfect Lawgiver. The laws of nature were considered an expression of His will. To admit that a law could change meant to recognize the imperfection or inconstancy of the Creator, which bordered on heresy.
2. *The mathematization of science.* Beginning with Galileo and Newton, science sought universal, mathematically rigorous truths. The ideal of “eternal formulas” (such as the law of universal gravitation) psychologically excluded the thought that the constants or rules themselves could evolve.

Only in the 18th and 19th centuries, with the weakening of church influence, did two figures emerge who dared to return the idea of historical development to science, and above all to physics: Denis Diderot and Friedrich Schelling.

Denis Diderot (1713–1784), in his works (primarily in *Thoughts on the Interpretation of Nature*), was the first in the Early Modern period to “biologize” physics, presenting the Universe as a living organism devoid of external control [16,17]. Diderot rejected the image of the clockwork Universe. For him, matter is in a state of eternal fermentation. According to him, what we call the “laws of nature” is merely a temporary order that arose from chaos as a result of accidental combinations of matter. Diderot argued that on the scale of eternity, the laws of nature have their “youth”, “maturity”, and “old age”. Just as animal species go extinct, physical regularities can also “die”, giving way to new ones. He suggested that if we could travel millions of years into the past or the future, we would discover a different physical reality. His views anticipated the modern idea that physical constants could have been different at the moment of the Universe’s birth.

While Diderot proceeded from matter, Friedrich Schelling (1775–1854) in his *Naturphilosophie* viewed Nature as a living, developing Spirit [18,19]. Schelling advanced a revolutionary idea: the laws of nature are not given to the Universe from the outside; they *emerge* in the process of its development. Nature evolves from simple forms (mechanics) to complex ones (chemistry, then organics). At each new stage, new laws arise that did not exist previously. For Schelling, a law is not a dead formula, but a “habit” of nature. As Nature (as a subject) becomes aware of itself and grows more complex, it can change its ways of acting. Schelling was the first to declare that nature has a *history*, and this history concerns not only the change of landscapes, but also the transformation of the very foundations of being. The world for him is an incomplete process, where the “rules of the game” are in constant becoming.

The works of Diderot and Schelling became a bridge between ancient intuition (Empedocles, Epicurus) and modern cosmology. They destroyed the myth of the “frozen Universe” and prepared science to accept the idea of global evolution, where even the most fundamental rules of nature can have a beginning and an end.

A crucial contribution to the topic discussed here was made by David Hume (1711–1776) and Immanuel Kant (1724–1804), who shifted the focus of attention from “nature” to how our consciousness perceives it. Hume approached the question as an empiricist: all our knowledge originates from experience [20]. But in experience, we never see “laws” or “necessary connections”. He pointed out the problem of induction (from the fact that the sun rose every morning, it does not logically follow that it will rise tomorrow) and the problem of causality (from the fact that event A constantly precedes event B, a causal connection between them does not follow with necessity). Our laws, therefore, are simply our habit of expecting the customary course of events. Since we do not observe a “necessary connection” in nature, there are no logical guarantees that the laws will remain the same tomorrow. The world can change at any moment without any contradiction.

Kant attempted to save science from Hume’s skepticism [21,22]. Kant argued that space, time, and causality are not properties of the external world, but “built-in filters” (categories, *a priori* forms) of our mind. According to Kant, we do not discover the laws of nature, but impose them upon it; our mind organizes the chaos of sensations into a coherent system thanks to the *a priori* forms existing within it. The laws of nature are the rules by which our mind constructs experience. We cannot see the world otherwise than as subjected to the law of causality, because causality is the “built-in software” of our consciousness. We know the laws only of the world we perceive (phenomena), but we will never know what “things-in-themselves” (noumena) are like. Can laws change according to Kant? For a human being—no; the laws of nature will seem immutable and universal to us, because our consciousness is structured precisely in this way (with categories of time, space, cause, etc.). But what would happen if we allow for the evolution of man and consciousness? The great philosopher did not consider this, but this question arose, and we will discuss it soon.

## 4. At the Turn of the 19th and 20th Centuries: Charles Peirce (1839–1914) and Émile Boutroux (1845–1921)

Until the mid-19th century, science relied on the image of the “Universe-as-a-mechanism”, where the laws of nature were considered eternal and immutable truths. Charles Darwin, in his work *On the Origin of Species* (1859), demonstrated that living nature is in a state of constant becoming—species are not eternal, they change. If the most complex part of nature, which is life, is variable, then it is logical to assume that more fundamental levels of reality—matter and its laws—could also have undergone evolution. C. Peirce became the first thinker who dared to transfer Charles Darwin’s theory of evolution from biological species to the very laws of physics [23,24,25]. He advanced an idea that was radical for his time: the laws of nature are not given from the very beginning; they “grow” and change.

For over 30 years, Peirce worked for the U.S. Coast and Geodetic Survey, conducting highly precise gravimetric measurements. It was precisely this work with “errors” that led him to the thought that ideal precision does not exist in nature. He concluded that an error is not always a human mistake and that the ideal of an “absolute law” exists only on paper, whereas nature always possesses a degree of “inexactness”, which serves as the engine of its evolution. Randomness is not a gap in our knowledge (as physicists of that time believed), but a real property of nature itself. At the foundation of the world lies a fraction of “primordial chaos” and freedom. It is precisely this spontaneity that allows the Universe to develop, rather than simply repeating the same cycles.

Peirce proposed a radical alternative to the concept of an “immutable law of nature”. He replaced it with the concept of “habit”. The laws of nature are not given to the Universe in a ready-made form. They are the result of a historical process. The Cosmos “learns” order. At the beginning of time, the laws were extremely “loose” and indefinite. Over time, under the influence of nature’s tendency toward uniformity, these random actions transformed into stable, “hardened” habits. If some event repeats accidentally, nature “remembers” it. To clarify, within Peirce’s framework of objective idealism, this cosmic memory and learning process do not presuppose a human-like, anthropomorphic consciousness. Rather, Peirce posits that ‘matter is effete mind,’ meaning that physical matter and the laws of nature are simply mind that has become hidebound by inveterate habits. The origin of this ‘memory’ lies in his metaphysical principle of synechism (continuity), which states that the universe is an interconnected, evolving semiotic continuum capable of retaining and generalizing tendencies over time.

According to Peirce, no law of nature is 100% accurate. There always remains a microscopic gap (“leeway”) for a random deviation, from which a new regularity may grow in the future. Peirce explicitly stated that the laws of nature continue to evolve, albeit extremely slowly, becoming increasingly rigid. Peirce called his philosophy “Darwinism in logic”. If Darwin showed that species are variable and evolve, then Peirce declared that the laws by which matter lives are also variable and have a history of development, learning order. If the laws of nature are not rigid and are “incomplete”, it means that human freedom can fit into the gaps between them.

Peirce’s contribution might look too metaphysical. However, 20th- and 21st-century science (from Heisenberg’s uncertainty principle to Lee Smolin’s theories of an “evolving Universe”) has confirmed Peirce’s intuition.

Speaking of Peirce, one cannot fail to mention his friend, the famous psychologist and philosopher W. James (1842–1910). It was he who made Peirce’s works known to a wide circle of researchers, while transforming his ideas and adding his own. James made a turn from Peirce’s ontology to epistemology and psychology [26,27,28]. He took Peirce’s central ontological idea that “law is a habit” and applied it to the human brain. James describes habits in Ref. [26] as “grooves” that are trodden into the nervous system. Just as Peirce’s Universe “gets used to” following the laws of physics, the human brain “gets used to” certain reactions. Peirce believed that the Universe evolves toward order. Over time, randomness becomes less frequent, and the laws become more rigid. James, on the contrary, believed that the world is fundamentally “unfinished” [27]. He did not believe in the inevitable triumph of a single order. For him, variability is an eternal property of the world, which always remains open to new forms and accidents. James believed that if belief in the variability of laws (or in God, or in free will) helps a person to live and act effectively, then this idea is “true” and useful. James thus “grounded” Peirce’s metaphysics. While for Peirce the variability of laws was a property of being that explained the diversity of the world, for James it became a condition for human action [28].

The French philosopher and historian of science Émile Boutroux (1845–1921) did not examine a changing world and its laws (things-in-themselves) as Peirce did, but studied the laws of the world that we extract ourselves while cognizing nature (phenomena) [29,30]. He presented his thoughts in his doctoral thesis (1874) [29]. In an era when classical physics was considered a “practically completed building” and scientists believed in absolute determinism (where the future is completely predictable from the past), Boutroux’s work became a true intellectual challenge. He declared that science takes for a “necessity” what is actually merely an “accidental superposition”. Boutroux’s key concept is contingency (from the Latin *contingentia*—randomness, non-obligatoriness). Boutroux shows that physical laws are not logically justified as strictly as mathematics. We cannot derive the law of universal gravitation from the laws of pure logic. In the chain of “cause and effect”, the effect always contains something new that cannot be 100% derived from the cause. He calls this microscopic gap contingency.

Boutroux argues that the world consists of strata (mathematics → physics → chemistry → biology → man) [30]. Each new level is “layered” upon the previous one, bringing with it new laws. The laws of the lower level do not dictate everything to the laws of the upper level. Physics cannot fully explain biology. Boutroux became a precursor to the idea of emergence—the appearance of fundamentally new qualities in a system that cannot be reduced to the sum of its parts. Thus, no matter how much we study the chemical composition of the elements of a living organism, there is no hint of its functions in them. Therefore, during the transition to biology, an emergent leap occurs: new laws are born that do not negate chemistry but are not derived from it either. As a result, the laws of nature evolve along with the complication of the world: in the young Universe, biological laws simply did not exist—they were “born” along with life itself.

The most important discovery of Boutroux is that laws can change not only at the birth of new levels of being, but also within already “established” domains, such as physics. These changes can be of two types:

1. *Change through the development of the world.* The world does not stand still, and those regularities that we record today can slowly change along with the global development of the cosmos.
2. *Change through the subject of knowledge.* Boutroux emphasizes the instrumental character of scientific knowledge. The laws formulated by science are not identical to “things-in-themselves”, but are representations of reality for human consciousness. An evolution of the cognitive abilities of the subject occurs, alongside the improvement of the analytical apparatus, allowing for the identification of deeper levels of reality. Thus, the “variability” of laws appears here as a consequence of human progress, which adapts its conceptual schemes to the unfolding complexity of a dynamic world.

Thus, Émile Boutroux demonstrated that a law of nature is not a divine decree, but a historically evolving category. The influence of Émile Boutroux on the scientific thought of the late 19th and early 20th centuries was immense precisely because he offered a philosophical justification for “scientific freedom”. His ideas exerted a major influence on the philosophy of H. Poincaré, A. Bergson, G. Bachelard, I. Prigogine, and many others.

At the turn of the 19th and 20th centuries, a final transition from ontology to epistemology occurred when considering the variability of the laws of nature. In the next three sections, these questions of the philosophy of science will be briefly examined.

## 5. Evolutionary Epistemology and the Variability of Laws

Traditionally, the laws of nature are considered either eternal truths or, if one follows Kant, immutable filters of the human mind. Evolutionary epistemology, which emerged in the second half of the 20th century, offers a different perspective: our conceptions of the laws of nature are cognitive adaptations. We perceive the world as ordered not because it is so “in reality”, but because such a vision helped our species to survive. We will briefly discuss here the two scientists whose contributions are most significant.

Konrad Lorenz (1903–1989) argued that Kantian categories (our “glasses” through which we see the laws) are not a mystical gift, but the result of natural selection [31,32]. Kant believed that the categories of space, time, and causality are given to us prior to any experience. Lorenz agreed but added: they are “*a priori*” for the individual, but “*a posteriori*” (derived from experience) for the species [31]. Lorenz maintained that our perception of causality is an “organ” just like an eye or a wing. It was formed during evolution for interacting with the mesocosm (the world of medium dimensions—the environment in which humans evolved). We see the world ordered in a specific way because ancestors who failed to see these regularities (for instance, the connection between the appearance of a predator and danger) simply left no offspring. If humanity had developed under the conditions of the microcosm (quantum reality), our basic laws of logic and physics would be different. For us, the laws of nature are an interface created by evolution to interact with reality.

While Lorenz studied the evolution of the brain, Karl Popper (1902–1994) applied Darwinism to scientific ideas themselves [33,34,35]. For Karl Popper, a scientific law is not a “truth carved in stone”, but a fortunate guess that has temporarily survived under the conditions of the severest testing. Popper completely transferred the Darwinian scheme of “variability—inheritance—selection” into the realm of logic and scientific method. He proposed a universal scheme through which any science develops:

P1 → TT → EE → P2 → …, (1)

where P1 is the initial problem, TT is the Tentative Theory, EE is Error Elimination, and P2 is the new problem.

Science begins not with observations, but when an old “law” or expectation ceases to work. We advance a bold, risky hypothesis (the analog of a genetic mutation). At this stage, we “invent” a new law. Next comes the comparison of the conclusions of this law with other laws and experiment, and the elimination of errors. This is the stage of “natural selection”. The theory is subjected to attempts at refutation through criticism and experiments. If the theory survives, it generates new questions, and the cycle repeats at a deeper level. Scheme (1) shows that knowledge grows not through the accumulation of facts, but through the correction of errors. We do not move toward a “final destination”; we move away from “errors”.

The evolution of the laws of nature in his understanding is not simply the replacement of one set of misconceptions by another, but a directed selection. Each new “mutation” of a scientific law must possess greater predictive power than its predecessor, making our knowledge increasingly deep and dense. The concept of “*verisimilitude*” was introduced by him in 1963 [33]. A theory possesses greater verisimilitude if the number of its true consequences is greater and the number of its errors is smaller.

Before Popper, a law was considered good if it could be confirmed (verified). Popper stated the opposite: anything can be confirmed (for example, astrology). A genuine scientific law must be falsifiable—it must clearly state which events are forbidden. Example: The law “All swans are white” is scientific because it is easy to refute with a single black swan. A law of nature is now always under the threat of refutation. We accept it only because it put itself in harm’s way (predicted a risky fact) and stood its ground. Thus, according to Popper, a “law of nature” is not an ultimate truth, but the fittest hypothesis that has not yet been refuted. The evolution of laws is a continuous process of the demise of less perfect theories and their replacement by better predictors.

Popper proposed a solution to “Hume’s problem” (which we discussed earlier). For Popper, laws are not conclusions from experience, but our active constructions. Since these are our guesses, they are by definition variable. We change a law as soon as nature says “no” to us.

Popper divided reality into three worlds: World 1: Physical objects. World 2: Our consciousness, where scientific hypotheses and theories, among other things, are born. World 3: The objective content of thought (theories, laws, problems). These are the products of World 2, somehow preserved in World 1 (in the form of books, scientific articles, etc.). The laws of nature “live” in World 3 and lead an independent life there: they develop, enter into contradictions, and generate consequences that the authors might not have even suspected. The evolution of laws is a process within World 3. We advance a theory (World 2), and it begins to “fight” with facts from World 1. If the theory loses, it “perishes” in World 3.

## 6. Social Epistemology and the Variability of Laws

Science is not created by isolated scientists. Cognition is fundamentally a collective process, and the study of this process is the subject of social epistemology. The laws of nature we operate with are the result of a complex social agreement and historically conditioned modes of thought. Here we will briefly focus on the contributions of three scholars.

The microbiologist and philosopher Ludwik Fleck (1896–1961) is considered a pioneer of social epistemology. In his 1935 work, he demonstrated that cognition is impossible outside of a group; it is not an individual process (as in Descartes or Kant), but a social one [36,37]. A scientist is never “one-on-one” with nature. Between the scientist and the object, there always stands a community that has taught them the language, the methods, and the ways of seeing the world. The thought style (*Denkstil*) is Fleck’s central concept. A thought style is a set of intellectual readinesses that makes certain facts “visible” and others “impossible” or ignored. For Fleck, a law of nature is not an absolute truth, but a “compulsory collective idea.” The thought style exerts pressure on the scientist: one simply cannot think otherwise while remaining inside the collective. Thus, for example, in the era of alchemy, the correspondence of metals to planets was considered a “law.” The scientists of that time were not foolish—it was simply that their “style” dictated exactly this interpretation of data. For Fleck, there is no “pure” nature. We always deal with nature passed through the filter of culture.

For Fleck, a “fact” or a “law” is not a piece of gold that a scientist finds in the ground. Rather, it is the result of a long sculpting process involving thousands of people, their errors, prejudices, and traditions. Here is how it works step-by-step. First, a vague idea about a problem exists in society. Scientists begin to investigate the matter. However, they do not look at the world with “clear eyes.” They possess a “thought style”—they look for what they are accustomed to finding. Researchers conduct experiments, argue, and make mistakes. Gradually, some regularity begins to emerge from the chaos of data. Those details that do not fit into the general picture are simply unnoticed by the collective (Fleck calls this “active blindness”). When the community of scientists reaches an agreement among themselves, a “scientific fact” is born. Within the collective, all data are fitted into the current style so skillfully that an illusion of absolute harmony and the truth of the law is created. The idea moves from the laboratory (where it was still doubted) into textbooks. Along the way, it is simplified, losing all its “buts” and “ifs.” As a result, for a student or a layperson, it is no longer a “hypothesis of a group of people,” but an “eternal law of nature.” A fact becomes “eternal” and “objective” only because people have stopped arguing about it. It is important to emphasize once again: (1) If we had a different “thought style,” we would sculpt a completely different “law” from the very same data. (2) A law is a social habit: we call something a “law of nature” simply because our collective has believed in it for so long that it has forgotten how the idea appeared in the first place. When an old thought style ceases to work effectively, the collective begins to experience discomfort. A shift in style occurs, and the “laws” are rewritten anew.

Thus, L. Fleck demonstrated that the “immutability” of the laws of nature is a socio-psychological effect. We believe in a law not because nature is unchangeable, but because our thought collective (*Denkkollektiv*) does not allow us to doubt our thought style.

Gaston Bachelard (1884–1962) is best known for his epistemological works of the 1930s [38,39]. Bachelard argued that the mind is not a *tabula rasa*. The most difficult thing in science is not to learn something new, but to forget the old. Our initial knowledge of the world is “sensory” and “naive.” Bachelard calls these primary images “epistemological obstacles” (*obstacles épistémologiques*). They reside very deeply within us. According to Bachelard, a change in the laws of nature occurs not when we find a new fact, but when we find the strength within ourselves to doubt the obvious. A law of nature is the result when we consciously cut away metaphors and feelings, replacing them with abstract models. Until the mind “breaks” the old habit of perception, a new law will not be discovered. For Bachelard, science is alive only as long as it is capable of denying its own dogmas and moving further into the realm of the “improbable.”

Bachelard categorically denied that science grows “brick by brick.” For him, the history of the laws of nature is a chain of intellectual revolutions. He introduced the concept of the “rupture” (*la rupture épistémologique*) to show that a new theory does not grow out of an old one; it refutes it. For instance, Einstein’s theory of relativity is not an “improved” Newton. It is a complete break with Newtonian concepts of absolute time and space. Bachelard demonstrates that there is no bridge between the laws of different eras. The mind must make a leap, a “mutation.” Therefore, the laws of nature are variable to us: they are merely temporary forms of equilibrium. As soon as the mind achieves a new rupture, the entire picture of the world is rewritten anew. Old laws do not disappear, but they cease to be the truth and become a “special case” or a “useful approximation” for a past level of development.

Bachelard asserts that a modern scientific object is a product of our instruments. We do not study the electron in nature; we study it in an accelerator. We do not study a cell; we study it by means of a microscope. An instrument is not merely a tool for observation; it is a “materialized theory.” Science is engaged in “phenomenotechnique” (*phénoménotechnique*)—it creates the phenomena it wants to study itself. Since laws “live” inside instruments, with the invention of each new tool, we essentially create a new nature. Since our instruments are constantly becoming more complex (from the telescope to the collider), it means that the “nature” we deal with is constantly changing. Consequently, the laws describing this artificially created reality change as well. This is Bachelard’s most radical idea.

The American historian of science Thomas Kuhn (1922–1996) started as a theoretical physicist, but upon engaging with the history of science, he discovered a strange fact: the great scientists of the past were not “under-educated”; they lived in completely different intellectual worlds. His 1962 book became a watershed: after it, science ceased to be perceived as a linear march toward truth [40]. Kuhn’s ideas resonate in many ways with the aforementioned ideas of Fleck and Bachelard. Some ideas he borrowed and developed, while others he arrived at independently. Kuhn’s central concept is the paradigm. This is not just a theory, but an entire complex of methods for a scientist (generally accepted theories and laws, methods for solving problems, and standards of what is to be considered scientific and what is not). Within a paradigm, the laws of nature seem eternal and self-evident. A scientist does not doubt them; they use them as tools. The paradigm defines the very reality in which the researcher lives.

According to Kuhn, the development of science consists of three main stages that cyclically replace each other. The first stage is the period of normal science. This is a period of tranquility. During this time, scientists do not look for new laws. They “fit” the world to the existing paradigm. If an experiment does not match a law, it is not the law that is to blame, but a poor instrument or a negligent scientist. This is a time of data accumulation. It is precisely here that a law of nature acquires its “immutability” through endless repetitions in textbooks and laboratories. The second stage consists of anomalies and crisis. Over time, facts appear that do not fit into the framework of the paradigm in any way (for example, the anomalous precession of Mercury for Newtonian physics). When there are too many anomalies, a period of ferment begins. Scientists begin to doubt the fundamentals. Laws cease to be perceived as “obvious,” and the search for new paths begins. The third stage is a scientific revolution. This transition from old laws to new ones is not a logical deduction, but a “gestalt shift.” Kuhn compares a scientific revolution to a political one. Old laws are not “refined”—they are overthrown. Scientists “before” and “after” a revolution live in different worlds. For a proponent of Newton, the size of an object is constant; for a proponent of Einstein, it depends on velocity. These are not different opinions about the same thing; these are different meanings of the very same words. Theories, according to Kuhn, are incommensurable. An Aristotelian physicist and a Newtonian physicist look at the very same pendulum, but they see different physical laws (one sees a body falling toward the center, the other sees an oscillation).

The evolution of physical laws is similar to biological evolution: it goes “from” simple forms to complex ones, but it has no ultimate goal. We change laws because a new paradigm handles current “puzzles” better, not because it “more correctly” reflects God or Nature. We choose those laws that allow our scientific community to work effectively at a given moment in history. Kuhn quotes Max Planck: “A new scientific truth does not triumph by convincing its opponents and making them see the light, but rather because its opponents eventually die, and a new generation grows up that is familiar with it” [7].

Thus, the development of science according to Kuhn is cyclical: from stability through crisis to revolution and back to stability. T. Kuhn’s work is considered the pinnacle of social epistemology.

## 7. Radical Constructivism and the Variability of Laws

While previous thinkers (Peirce, Popper, Kuhn) in one way or another left room for the “objective world,” radical constructivists take the final step: they assert that the laws of nature are 100% constructions of our brain, having no direct analogues in reality. Here we will discuss the most prominent representatives of this direction.

Jean Piaget (1896–1980) was a biologist by training, and this defined his approach: he viewed the development of intelligence as a biological adaptation of the organism to the environment. His research on children became the foundation upon which radical constructivism later grew. Piaget himself defined his approach as genetic epistemology. Piaget’s main idea is that the laws of nature are not “imprinted” onto the child’s brain from the outside; the child actively constructs them through action. In other words, the laws of nature are not “discovered” in a ready-made form; they are constructed by the mind in the process of its adaptation to the environment [41,42,43].

Piaget explained the modification of our internal “laws” (schemata) through assimilation and accommodation. In assimilation, a child attempts to fit a new experience into old frameworks (for example, a child knows the law “everything round is a ball” and calls an orange a ball). Accommodation occurs when an experience does not fit into the old frameworks, and the brain is forced to change the frameworks themselves (the child realizes that an orange is not a ball; it is sticky and it is eaten; a new “law” or category is created). According to Piaget, the evolution of science is a continuous accommodation of humanity. When the data of the microcosm did not fit into Newton’s laws, physics made an “accommodation” and created quantum mechanics [43].

Laws as “Intellectual Equilibrium” (Equilibration) is Piaget’s key concept, explaining both the stability and variability of laws. A law of nature is not an absolute truth, but a state of maximum stability of the mind in its relations with the world. As long as a law allows us to successfully predict events (to assimilate experience), we are in equilibrium. The appearance of anomalies in science causes “cognitive friction.” The old law can no longer compensate for external perturbations. The equilibrium collapses. Piaget argues that development is a transition from a “fragile” equilibrium to a more “mobile and stable” one. Einstein’s laws are “better” than Newton’s laws not because they are “truer,” but because they represent a higher level of intellectual equilibrium, capable of encompassing a much larger volume of contradictory facts [43].

Piaget demonstrated that logical and mathematical laws grow out of physical actions. First, a child touches and moves objects (sensorimotor intelligence). Then, they learn to replace actions with symbols. Only after that can they operate with “laws” in the mind. At this stage, the adolescent (and the scientist) learns to separate the form of a law from its content. It is no longer important what exactly is moving—they see the invariant structure (the formula).

Piaget’s experiment on “Object Permanence” is a foundational study that formed the basis of constructivism [44]. The essence of the experiment is as follows: an infant under 8 months old is shown a toy, which is then covered with a cloth. The child behaves as if the toy has ceased to exist and does not look for it. Conclusion: The law “things exist even if I do not see them” is not innate. The child must “construct” this law through manipulations with objects. If even such a basic law of reality is a construction, then all other laws of physics are all the more so.

For Piaget, the laws of nature are dynamic tools for coordinating our experience, stages of intellectual equilibrium. They are variable because our mind strives for a more perfect equilibrium. They are constructive because they are born from our actions, rather than from passive observation. They are hierarchical: each new “law” is a step toward a deeper and more universal understanding of the unity of the world.

Ernst von Glasersfeld (1917–2010) transformed Piaget’s psychological insights into a powerful philosophical system. It was he who coined the term “radical constructivism” to emphasize that we do not merely “refine” our knowledge of the world; we break completely with the idea that knowledge can reflect objective reality [45]. Von Glasersfeld’s key idea is the proposal to replace the concept of “truth” (the correspondence of knowledge to the world) with the concept of “viability” (the correspondence of knowledge to our goals). Knowledge is not acquired passively; it is actively constructed by the cognizing subject. The function of cognition is adaptive and serves to organize the experiential world. A metaphor can be provided here: knowledge is a key that we individually manufacture ourselves. The key fits the lock not because it “resembles” the interior of the lock, but because it allows us to open the lock. We will never know the structure of the lock (reality); we only know that our key (the law) worked. Drawing on biology, von Glasersfeld argued that cognition is not a search for “photographs” of reality, but a means of survival. Our mind constructs models of the world solely to exist successfully within it and to avoid obstacles. Von Glasersfeld insisted that our consciousness can never step “outside” to compare its picture with the external, surrounding world. We are locked within our experience. Therefore, any talk of “objective laws” is a metaphysical illusion.

If the laws of nature are our “keys,” then their modification is a natural process of refining our instrumentation. When an old law (key) ceases to “open the doors” of experience (for example, fails to explain new data in physics), we construct a new, more viable one. By formulating a new law, we coordinate our experience so that it becomes predictable and we remain in cognitive equilibrium. A change in laws is not an approximation to a hidden truth, but a continuous process of inventing increasingly effective ways to live in a world that forever remains a “black box” to us. Since each individual builds their own world, “generally accepted laws” are the result of their social coordination.

Continuing von Glasersfeld’s thought that knowledge is merely a “key” to reality, Maturana and Varela ask: what exactly in our biology compels us to create such keys? The contribution of Humberto Maturana (1928–2021) is associated with his classical neurophysiological experiments on animal vision. Investigating how the eye of a frog or a pigeon transmits a signal to the brain, he demonstrated that the central neurons of the visual system react only to electrical perturbations coming from neighboring neurons [46]. The signal from the retina is not a “piece of reality,” but merely a perturbation (a shove) that forces the nervous system to change its internal state. The brain reacts only to itself; it is an “operationally closed” system. Our cognition is not a description of external objects, but a process of maintaining the internal electrochemical stability of the organism in response to external stimuli. Maturana uses the metaphor of a captain in a submarine without portholes [47]. The captain has only instruments (neuronal impulses). He has never seen the ocean (the external world) and merely coordinates the positions of the instrument needles within specific ranges.

Francisco Varela (1946–2001), a student of Maturana, sought to find measurable biological correlates of how our brain “brings forth” its world [48]. Most famous are his studies on neural synchronization in the late 1990s, where he attempted to understand how disparate signals in the brain assemble into a single “law” or “image” [49]. Varela used multi-channel EEG to record the brain activity of people who were shown “ambiguous” contrast images (for example, Mooney faces hidden in chaotic smudges). He discovered the phenomenon of phase synchronization. At the exact moment when a person “recognized” the object (the moment of enaction, the creation of meaning), different areas of the brain began to vibrate at the same frequency (gamma rhythm) [49]. Thus, a “law” or an “object” is a dynamic event within the neural network itself [50]. Cognition is a rhythmic dance of neurons that momentarily “freezes” into a stable structure. The modification of laws is a natural process of retuning this harmony. We change laws because we ourselves change in the process of life, constantly creating new worlds and new ways to be stable within them.

Modern neuroscience has integrated the ideas of constructivism, transforming them from philosophical concepts into testable models of brain function [51]. The notion of the brain as a passive receiver of information has been replaced by the model of predictive processing (active prediction): the brain does not copy reality but constantly constructs it, advancing hypotheses about the causes of sensory signals. In this context, our perception is defined as a “controlled hallucination” (a term popularized by the British neurobiologist Anil Seth) [52]. The essence is that the brain is locked inside the skull and receives only electrical impulses, not reality itself. To understand what is happening outside, it generates its own picture of the world (“hallucinates”), and uses incoming signals from the sense organs merely to correct errors in this picture in a timely manner [52]. If the hallucination successfully matches the signals of the environment, we call it reality; if the connection is lost, illusions or psychosis arise.

Today, the legacy of constructivism develops along several lines [51]. (1) Refinement of mechanisms: it is investigated how neural networks hierarchically organize experience, turning a stream of random signals into stable “laws.” (2) Generalization through learning: it is studied how innate mechanisms of perception interact with culture, allowing the brain to complete models of objects that we do not see directly. (3) Functional approach: the search for regularities is viewed as a tool for adaptation. The brain identifies the rules of the surrounding world to make its “hallucinations” maximally useful, including energetically, for survival.

To summarize: (1) We cognize the world not to know the “truth,” but to exist within it. (2) A law is a plastic interface; it is inherently variable, as it is part of a living, pulsating system that constantly re-creates itself. (3) The modification of the laws of nature is proof of our creative freedom. We are not slaves to pre-established laws; we are their authors, constantly rewriting the “code” of reality for the sake of new horizons of experience.

## 8. I. Prigogine and L. Smolin

From the preceding discussion, one might form the impression that it is mainly philosophers and biologists who are interested in the variability of our laws and our conceptions of them. Here we present the views of two well-known physicists regarding the issue under consideration.

Ilya Prigogine (1917–2003) repeatedly spoke out against the view of the world as a reversible mechanism, which had dominated since the time of Newton. In classical physics, laws were considered deterministic and time-symmetric (the future and the past are logically equivalent). Prigogine called this the “denial of time.” In his work [53], he writes: “Nature is indeed related to objective laws, but these laws no longer express certainties; they express possibilities.”

I. Prigogine and his like-minded colleagues (the so-called Brussels scientific school) demonstrated, through numerous examples, that far from equilibrium, during the exchange of energy and matter with the surroundings, the emergence of so-called dissipative structures is possible. Examples include Bénard convection cells, turbulence in fluids, dendritic ice crystals (snowflakes), periodic chemical reactions (the Belousov–Zhabotinsky reaction), etc. According to Prigogine, while everything in equilibrium tends toward chaos (the growth of entropy), far from equilibrium irreversibility gives rise to complexity, organization, and even life. Such irreversible processes in physicochemical systems can be viewed as a source of novelty.

Prigogine’s main innovation lay in the assertion that the direction and irreversibility of time (the arrow of time) are not an illusion, not a statistical consequence, and not a result of the initial conditions of the Universe, but a fundamental property of nature itself. Prigogine believed that existing reversible equations are incomplete and crude. He argued that the laws of modern physics are applicable only to idealized, simple systems, whereas in the complex, real world, irreversibility dominates, and consequently, more complex equations are required for its description.

“Time is not just the parameter of decay. Time is the creator of order.” [1] In this context, time ceases to be a simple parameter or dimension; it becomes an active agent of becoming, a process of creating complexity and novelty in nature. The real world is open, unstable, and unpredictable. “We are at the end of a physics that describes a static, timeless universe. We need a physics that incorporates irreversibility at its very foundation.” [53] Prigogine proposed a mathematical restructuring of physics and introduced a “time operator” into quantum mechanics [54]. The goal was to embed the arrow of time into the very foundations of physics. However, this program did not receive wide recognition in the physics community because his “irreversible quantum mechanics” did not yield new testable effects. At the same time, Prigogine’s ideas and numerous books on dissipative structures and time have exerted and continue to exert an immense influence on scientists, especially physicists, chemists, and biologists.

One of Prigogine’s profound conceptual shifts was the revision of the concept of entropy. Traditionally, starting with L. Boltzmann, entropy was viewed as a measure of our “ignorance” regarding the microscopic details of a system. Prigogine rejected this “anthropocentrism.” For him, entropy and the irreversibility of time are objective, fundamental properties of nature, rather than a result of human limitation or a “lack of information” [53]. Prigogine viewed entropy as a measure of the internal randomness ontologically inherent to a system (let us note that, mathematically, this definition of entropy is completely consistent with Boltzmann’s approach). In his understanding, the higher the entropy in an open system, the higher its capacity for self-organization at bifurcation points.

Prigogine was a successor to the lineage of “variability”; he was well-acquainted with and cited the works of C. Peirce and É. Boutroux. According to Prigogine, the laws of nature possess a historical character. The world is not “given” once and for all; it is in a process of becoming.

The second physicist to be briefly discussed here is Lee Smolin (1955). He is one of the few well-known contemporary theoretical physicists who explicitly states that physics has reached a dead end precisely because of the belief in the immutability of laws. His arguments are built at the intersection of cosmology, philosophy, and biology. His main theses are set forth in works [55,56].

According to Smolin, modern physics is sick with mathematical Platonism, i.e., the belief that laws exist in an eternal, extra-spatial, timeless world of mathematics, and this prevents us from understanding why exactly these laws govern the Universe. According to Smolin, *to explain why the universe is as it is, we must accept that time is real and that the laws of nature may evolve in time* [56].

The most interesting idea of Smolin, which he substantiates with his theoretical calculations, is the hypothesis of cosmological natural selection [55,57]. The essence of the hypothesis is that *universes reproduce by means of black holes, which give rise to new universes in their interiors. Each new universe has slightly different* fundamental constants and laws, *and those that produce more black holes have more offspring* [55,57]. As a result, over time, universes with laws optimal for the formation of the largest number of black holes are selected. Interestingly, since according to the foundational works of J. Bekenstein [58] and R. Penrose [59] on gravitational thermodynamics, black holes are absolute record holders in entropy production, Smolin’s ideas naturally lead to the maximum entropy production principle—a principle that forms the foundation of the modern physics of irreversible systems [60,61,62].

Thus, while traditional physics assumes that the laws governing the Universe (for example, the laws of Newton or Schrödinger) have been identical for billions of years and will remain unchanged in the future, Smolin rejects this “dogma.” He views physics as a historical science: “*The universe is a process, not a thing. And like all processes, it unfolds in time*” [56].

In his critique of the traditional approach, Smolin also relies on Leibniz’s principle of sufficient reason: every fact must have an explanation as to why it is exactly this way and not otherwise. If the laws are eternal and immutable, we will never be able to explain why the constants (for example, the speed of light) have precisely those values. They are simply “given.” The only way to make the laws of nature explainable is to recognize that they are the result of a process, that is, evolution in time [56].

The main point of criticism against the ideas put forward by scientists like I. Prigogine and L. Smolin is as follows: why change the paradigm, ignoring the successes of general relativity and quantum mechanics, if everything works well? And since the standard paradigm is timeless, one must accept it. However, the following counterargument can be made. If one adheres to such a point of view, then Ptolemy’s paradigm regarding the rotation of all celestial bodies around the Earth should also have been left uncriticized and ultimately unreplaced. After all, as is well known from the history of science, Ptolemy’s approach excellently predicted astronomical events mathematically, and initially did so significantly better than the newly emerged heliocentric paradigm of Copernicus and Galileo [56,63,64].

## 9. The Evolution of Laws and the Development of Science

As follows from the preceding discussion, science develops continuously; new laws are discovered and already existing ones evolve. However, the currently available algorithm of this process, according to works [10,11], raises certain questions. We present here the main essence of the critique presented therein, which was based on the analysis of the development of physics—one of the most highly developed natural sciences to date.

**Figure 1:**
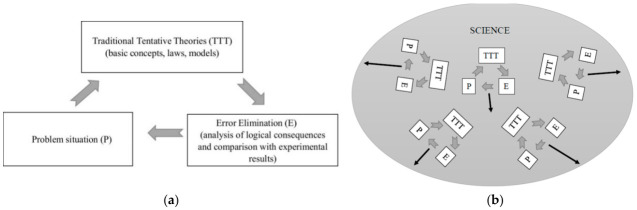
The evolution of traditional tentative theory (**a**) [11] and science (**b**). The traditional method.

When building a particular branch of science, a certain Tentative Theory is usually formulated, within which one can always distinguish three components: (1) basic concepts, (2) laws connecting these basic concepts, and (3) a set of assumptions and idealizations (a model). The analysis of the consequences of a Tentative Theory, especially when comparing them with experience, leads to the emergence of a number of problems, which in turn lead to the adjustment and modification of the Tentative Theory (see Figure 1a). During such adjustments, modifications can occur in all three parts of the theory. After a series of similar iterations, the theory takes a more or less finalized form (from the viewpoint of the majority of researchers) for a sufficiently long period, until some new “unexpected” problem arises. This problem leads to a new and sometimes substantial modification of the Tentative Theory—resulting in a so-called scientific revolution. This has been discussed in the previous sections. Although historical evidence from the development of science and technology shows this approach to be effective and fruitful, methodologically, such a development of a theory has its drawbacks. We present them below.

First, basic concepts (the “language” of a theory) are usually not defined in any way initially (neither by means of operational measurement nor by cognitive operations). They are introduced intuitively. This is particularly evident in the case of such a vital quantity in mechanics as time. From traditional expositions, it is absolutely unclear how time is to be measured or introduced without relying on the laws of mechanics that were already derived under the assumption that time exists and possesses a number of properties.

Second, theories as a whole (elements of all three parts) are “fitted” to experimental data. Thus, basic concepts, laws, and models essentially share a single origin and a single means of validity control—the experiment. These components form a unified system capable of describing a certain natural phenomenon as a whole, evolving according to the scheme in Figure 1a. During iterations, it becomes possible to relatively freely modify almost any part of the Tentative Theory. This results in a vast and unquantifiable number of degrees of freedom during such optimization. All this is further aggravated by the fact that when comparing with an experiment, its results are frequently treated and interpreted within the framework of the current, not yet finalized Tentative Theory. The outcome of everything listed above is a loss of confidence in the uniqueness and fundamental nature of such theories. Despite the conclusion drawn here, historically it is found that basic concepts and laws (principles) are often ascribed a significance that significantly exceeds the boundaries of the Tentative Theory within which they were formed. For example, one attempts to utilize the laws of thermodynamics in cosmology. It is obvious that, given origin of tentative theories, such an “extrapolation” can turn out to be either highly bold and fruitful, or adventurous and erroneous.

Third, different Tentative Theories investigating distinct, often distant fields of science (not necessarily limited to the natural sciences) may share similar basic concepts. However, each of these Tentative Theories develops independently of the others (see Figure 1b). As a result of iterations and optimization within each theory, basic concepts may acquire increasingly different properties. This leads to the fragmentation of science into weakly interacting parts, the loss of the universality of scientific knowledge, and an even greater loss of mutual understanding among scientists of different specializations. All of some undoubtedly reflects negatively on the progress of science as a whole. Thus, for example, the concept of time in science has come to possess different properties not only among biologists or historians, but even among representatives of different branches of physics, such as thermodynamics and classical mechanics.

**Figure 2:**
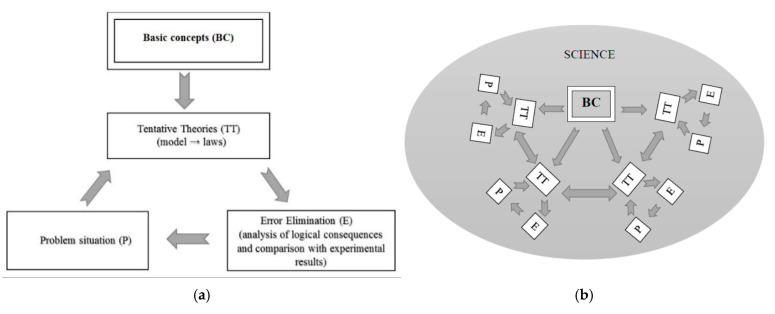
The evolution of tentative theory (**a**) [11] and science (**b**). The proposed method.

A possible methodological way out of the current situation was previously proposed in works [10,11]. We outline it here following work [11]. Basic concepts (for instance, time) must be formulated in such a way that they can be defined (measured, calculated) in a uniform manner for any field of modern science (including the humanities). Further, moving on to the study of a particular phenomenon of the surrounding world within the framework of a certain Tentative Theory, a specific model is constructed (a certain set of axioms, idealizations, and assumptions). After that, using the introduced basic concepts and the model, laws (consequences) are derived deductively, which are then compared with experience and analyzed (Figure 2a). In the event of finding certain discrepancies and problems, the model is adjusted, and the whole process is repeated. At the same time, the attitude toward basic concepts remains extremely conservative. These concepts affect and bind together the entire framework of science with its numerous models; i.e., they form the basis of our scientific picture of the world (Figure 2b). It should be noted that, of course, fundamental concepts can be modified, although only as a last resort and with extreme caution.

Thus, the main differences between the new scheme and the old one are as follows:

- Basic concepts are introduced in an explicit, constructive, and uniform manner. This is precisely what allows us to state that these concepts are basic.
- Laws are the logical consequence of the deductive development of basic concepts by means of models. Thus, a strict hierarchy exists: basic concept + model → law.
- Optimization during analysis and comparison with experiment is applied solely to the propositions of the model. As a result, the number of degrees of freedom when analyzing potential discrepancies between the tentative theory and observations is sharply reduced.

Such a hierarchical scheme allows science to develop as a unified framework in which individual directions do not move away from each other (Figure 1b), but rather complement each other through interaction (Figure 2b). The evolutionary benefit lies in the mutual enrichment and advancement of various scientific fields through this interaction (via shared foundational concepts). The isolated development of fragmented theories represents a highly “fragile” strategy, as is well known in evolutionary biology.

This approach is as yet unfamiliar to physics. Nevertheless, certain similar ideas have been expressed and implemented previously [11,12,65].

## 10. Entropic Measure of Time and the Evolution of Laws

As follows from the previous section, basic concepts are the central and most important link in the development of science. The purpose of this section is to give an illustrative example of deriving a law (based on the Basic concept and model), which is a very unconventional approach for physicists. Among basic concepts, time is one of the most significant, since the description of any real process is impossible without it. In works [10,11,12], the so-called Entropic Measure of Time (EMT) was proposed, according to which the change in time Δ*τ* and the change in entropy within the system Δ*S* are considered proportional to each other:

Δ*τ* ∝ Δ*S* (2)

Here, entropy *S* is connected in a traditional manner with the logarithm of the number of microstates corresponding to a certain macrostate whose time is being measured. The origin of these microstates can be attributed to randomness as ontologically inherent to both the world and the observer (i.e., in the spirit of I. Prigogine), or due to our ignorance or the coarsening of reality (i.e., in line with the classical Boltzmannian approach). In the general case, entropy is understood in the most general, information-theoretic sense; however, for many practically important cases, it can be considered thermodynamic [10]. Metric (2) possesses two important properties that distinguish it from traditional metrics [10]. First, it is universal, as it can be introduced for any real system in which any changes occur. Second, such an operationally introduced metric allows, if necessary, for the deductive derivation of the laws by which the system develops. This is precisely what enables the realization of the scientific development scheme presented in Figure 2. An example of deriving the laws of mechanics from metric (2) was presented in work [11]. As follows from works [9,11,12], the relationship between EMT *τ* and classical astronomical (mechanical) time *t* is frequently logarithmic, i.e.,

*τ ∝ ln t.* (3)

As evident from (3), EMT is non-uniform. It can be seen that the proposed framework does not reject other traditionally used time measures. Within the scope of the respective scientific discipline, different historically utilized measures of time can always be linked to EMT.

Since the approach presented in Figure 2 is non-standard, let us illustrate how it works using the simplest example. Suppose we want to describe the motion of particles under thermal equilibrium. We choose the coordinate as the basic characteristic, and as the mathematical model, we select particle collisions, the coordinates of which obey the central limit theorem. As a result, the distribution function of the particle deviation *x* is *f*(*x*) ∝ exp(−*x*^2^/2σ^2^)*,* where *σ*^2^ is the variance of the deviation. Let us assume that the system entered the state under consideration as a result of a non-equilibrium process in which the initial state corresponded to absolute zero. Assuming that the initial state corresponds to the zero moment of time (in any of the time scales), according to (2), we obtain that:

(4) $$\tau \propto \;S=-\int_{-\infty }^{\infty }f\left(x\right)ln\;f\left(x\right)dx=ln\sigma +const$$

According to (3), *t = σ*/*α*, where *α* is some constant. Substituting this relationship between variance and time into *f*(*x*), we obtain as a result the classical Maxwell distribution function for the particle velocity *υ*: *f*(*υ*) *∝* exp (−*υ*^2^/2α^2^*)*. Since the relationship between *τ* and *t* is linear for small times, the form of the distribution will have the same appearance for both times in this region. Thus, based on the simplest model and EMT, the velocity distribution law under thermal equilibrium has been obtained. A substantially more interesting, theoretically important, and non-trivial derivation of the laws of mechanical motion of particles, including their interaction law, is provided in work [11].

As stated above, a human being constantly constructs models of reality in everyday life, and this process has genetic, age-related, and social roots. The construction of laws by scientists occurs in an analogous way (here, the social, collective aspect is merely more substantial than the individual one). The world around us evolves; we, as a part of it, also evolve and change. In the process of our development, we create tools that change the world and allow us to see the world differently (at different scales, wavelengths, etc.). All this leads to the fact that our models of the world will change. Applying EMT to them, we will obtain new laws according to the scheme in Figure 2. Whether a new law emerges due to ontological or epistemological reasons is a less significant question, since laws, according to all the aforementioned, do not claim to be the truth, but merely contribute to the success of our interaction with reality.

## 11. Conclusions

It is pointless to distinguish between the change in nature itself (its laws) and our descriptions of it (our laws). A truth expressed by someone is never final; together with objective reality, we “create” truths. From this, two of its characteristics follow: (1) truth is variable, and (2) truth depends on the conceptual framework into which we place it. As the analysis conducted in this work has shown, the laws of nature are variable for several reasons:

- Since the organism and the environment constantly influence each other, the structure of our nervous system slowly changes. When the structure of the “calculator” (the brain or instruments) changes, the laws “calculated” by it change as well.
- Laws are not the “truths of matter,” but parameters of our interface of interaction with the world. When humanity invents a new tool (for example, a collider or a quantum computer), it creates a new domain of reality that requires entirely new laws.
- The modification of laws occurs when the old “rules of the game” no longer allow a biological or social system to maintain equilibrium in an increasingly complex and evolving environment.

The variability of laws is evidence of the creative plasticity of life. It is vital that science, while evolving together with its laws, does not fragment into weakly interacting parts, as can occasionally be observed today. A methodological solution that allows growing knowledge to remain unified has been presented in this review. The Entropic Measure of Time (EMT) serves as an essential binding element of this entire continuously constructed and constantly modified temple of science.

## Data Availability

No new data were created or analyzed in this study. Data sharing is not applicable to this article.
